# Utility of carboplatin therapeutic drug monitoring for the treatment of neonate and infant retinoblastoma patients in the United Kingdom

**DOI:** 10.1038/s41416-024-02728-1

**Published:** 2024-06-13

**Authors:** Gerard C. Millen, Alice Lawford, Catriona Duncan, Helen Jenkinson, Gareth J. Veal, Shelby Barnett

**Affiliations:** 1 Department of Paediatric Oncology, Birmingham Children’s Hospital, Birmingham, UK; 2 Department of Paediatric Oncology, Great Ormond Street Hospital, London, UK; 3 Translational & Clinical Research Institute, Newcastle University Centre for Cancer, Newcastle University, Newcastle upon Tyne, UK

**Keywords:** Eye cancer, Paediatric research, Pharmacokinetics

## Abstract

**Background:**

Retinoblastoma is the most common intra-ocular malignancy in children and frequently presents in very young patients who commonly require intravenous carboplatin. Delivering this is challenging due to a lack of uniform dosing recommendations, rapid changes in physiological function and the risk of side-effects.

**Methods:**

We conducted a retrospective review of neonates and infants in the UK with retinoblastoma, who have undergone carboplatin therapeutic drug monitoring (TDM). We report on the pharmacokinetic, treatment efficacy and toxicity data.

**Results:**

In total, 29 patients (median age 5 weeks at treatment onset) underwent a total of 74 TDM guided cycles of chemotherapy, involving real time sampling and dose adjustment. An additional 13 patients underwent TDM sampling to modify doses between cycles. Without the adoption of TDM guided dosing, carboplatin exposures would have been ≥20% outside the target AUC in 38/78 (49%) of treatment cycles. Excellent responses and a reassuringly low incidence of toxicities were observed following dose adjustment, despite the young patient age and the implementation of dose increases in the majority of cases.

**Conclusions:**

Real time TDM is safe, effective and deliverable for neonates and infants receiving carboplatin for retinoblastoma and should be considered standard of care up to the age of 6 months.

## Background

Retinoblastoma is the most common primary ocular tumour in childhood affecting 1 in 15,000–20,000 children, equating to approximately 50 children per year in the UK [1]. Recent advances mean that diagnosis is even possible in utero [2]. Treatment depends on the stage of the tumour and can include a conservative approach with focal treatment (laser photocoagulation, cryotherapy, plaque brachytherapy), systemic or targeted chemotherapy, or primary enucleation [3]. Targeted chemotherapy includes chemotherapy delivered directly into the ophthalmic artery (IAC), as well as chemotherapy delivered to the vitreous (intravitreal chemotherapy) or the anterior chamber (intra-cameral chemotherapy). Patients receiving systemic chemotherapy are at risk of myelotoxicity requiring blood or platelet transfusions, ototoxicity, second malignant neoplasms and potential long-term nephrotoxicity amongst others [1, 4–14].

A significant proportion of children diagnosed with retinoblastoma will be less than three months of age, with approximately 10% of all retinoblastomas and up to 70% of familial retinoblastomas presenting in the first 28 days of life [15]. For this cohort of patients, if chemoreduction of the tumour is necessary, treatment with systemic intravenous chemotherapy is usually indicated rather than IAC [16]. This poses a particular challenge due to the significant physiological changes that take place within the first few weeks of life and a limited understanding of drug disposition at this early age. The challenges associated with treating neonatal and infant patients with cancer have recently been reviewed in detail and highlighted as an important area for further research [17, 18].

Traditional recommendations for dosing chemotherapy in younger children with retinoblastoma have included either giving a percentage dose reduction based on body surface area, with the youngest children receiving the lowest dose, or dosing based upon weight (mg/kg), however there is no defined standardised approach [11, 16]. As a result, much controversy exists regarding the achievement of optimum therapeutic dosage and the safety of increasing the dose of carboplatin due to the trepidation of increased toxicity.

Real-time therapeutic drug monitoring (TDM) enables the clinician to have feedback on the distribution of exposure to a defined medication. This allows for dose adjustments with the hope of providing an efficacious dose of chemotherapy, whilst minimising the potentially life-altering side effects such as ototoxicity, which has been reported in 0–25% of patients exposed to carboplatin [6–10, 19].

The current study was conducted to better understand this relationship and determine any apparent short term and long-term side effects in our treated patients, to help provide evidence towards guiding clinical practice. Herein we report on the UK experience of using TDM in neonates and infants with retinoblastoma.

## Methods

### Study design and population

The technique of carboplatin TDM in a retinoblastoma setting was widely introduced in the UK in 2006. All children with retinoblastoma who received systemic chemotherapy including carboplatin (either as a single agent or in combination with etoposide and vincristine) and underwent TDM were included in this study. This included patients enroled in a formal clinical trial (ISRCTN 10139334) or who had TDM as part of standard clinical management between September, 2006 and December, 2021. Patients who underwent TDM were identified from the patient database at the Newcastle University Centre for Cancer (NUCC). These data were cross-referenced with the retinoblastoma databases at Birmingham Children’s Hospital (BCH) and Great Ormond Street Hospital (GOSH), the two primary centres coordinating care for children with retinoblastoma in the UK.

### Data collection

Data reporting on TDM including patient age, weight, dose delivered, creatinine clearance and area under the concentration time curve (AUC) were obtained from the NUCC. Corresponding clinical data included; incidence of nephrotoxicity (based on repeat serum creatinine levels, chromium-51 labelled ethylene diamine tetraacetic acid (^51^Cr-EDTA) [20] measured GFR or cystatin-C [21]), incidence of ototoxicity (not all patients had repeated assessments), need for blood product support, episodes of febrile neutropenia and efficacy (need for second line therapies). These data were obtained from the respective databases at BCH and GOSH, with review of individual patient case notes conducted where necessary. The project was registered as an approved service evaluation in both BCH and GOSH.

### Blood sampling and analysis

For patients undergoing carboplatin TDM dosing, a total of three 1 ml blood samples were routinely obtained from a central line, one taken mid-infusion, a second sample at the end of infusion and the final sample collected 1–2 h after the end of the drug infusion. Plasma was obtained from whole blood samples by centrifugation (1200 g, 4 °C, 10 min), and 0.5 ml removed and placed in an Amicon Centrifree micropartition unit with a 30,000 MW cut-off (Millipore, Edinburgh, UK). This sample was centrifuged (1500 g, 4 °C, 15 min) to obtain plasma ultrafiltrate for determination of free carboplatin levels. Samples were sent by overnight courier from clinical centres around the UK, on dry ice and in an insulated container, to the NUCC. Platinum pharmacokinetic analyses were carried out by flameless atomic absorption spectrophotometry (AAS) using an Analyst 600 graphite furnace spectrometer (Perkin-Elmer Ltd, Beaconsfield, UK) as previously described [22, 23].

### Therapeutic drug monitoring

Carboplatin clearance and AUC were determined by Bayesian analysis using a 2-compartment model as previously described [22–24]. For patients being treated on a 3-day carboplatin schedule, dosing was routinely adjusted on day 3 of treatment, based on the drug exposure and clearance values determined on day 1, to achieve the desired target cumulative AUC. Dose adjustments were recommended for patients with day 1 AUC values > 10% outside the target daily AUC and were calculated based on the actual carboplatin clearance determined on day 1, and the remaining AUC required to achieve the target cumulative exposure. Carboplatin AUC values of either 5.2 mg/mL.min or 7.8 mg/mL.min were targeted over 3 days of treatment as determined by the treating clinician and based on individual patient characteristics as well as the observed response to treatment on previous cycles of treatment (where relevant). Initial dosing on day 1 of subsequent cycles of carboplatin treatment was commonly guided by the exposures observed on cycle 1, with TDM again carried out over the 3 days of treatment. For all patients receiving carboplatin over a single day, basic clinical information was collected in addition to observed carboplatin clearance.

## Results

### Patient demographics

A total of 29 patients received adaptive carboplatin dosing over 3 days of treatment across 74 cycles of treatment. The study population had a median age of 5 weeks at the time of their first cycle of chemotherapy (range 9 days to 6 months). The median body weight (BW) was 4.1 kg (range 1.6–6.3 kg) and median body surface area was 0.26 m^2^ (range 0.14–0.34 m^2^). A summary of patient characteristics is provided in Table 1. An additional 13 patients had sampling following a single dose of carboplatin. This group had a median age of 22 weeks at the time of their first cycle of chemotherapy (range 13 weeks to 21 months). The median follow up time from the end of systemic chemotherapy is 4 years (range 4 months to 13 years).

**Table 1:** Summary of patient characteristics.

Characteristic	Number (%)
Evaluable patients	29
Age at start of treatment (weeks)	
0–4	12 (41)
5–8	9 (31)
9–12	4 (14)
>12	4 (14)
Sex	
Male	15 (52)
Female	14 (48)
Body weight (kg) at first cycle	
Median	4.1
Range	1.6–6.3
Body surface area (m^2^)	
Median	0.26
Range	0.14–0.34
Laterality of tumour	
Unilateral (non-genetic)	8 (28)
Unilateral (genetic)	3 (10)
Bilateral	18 (62)

### Carboplatin treatment and dose adjustment

**Fig. 1:**
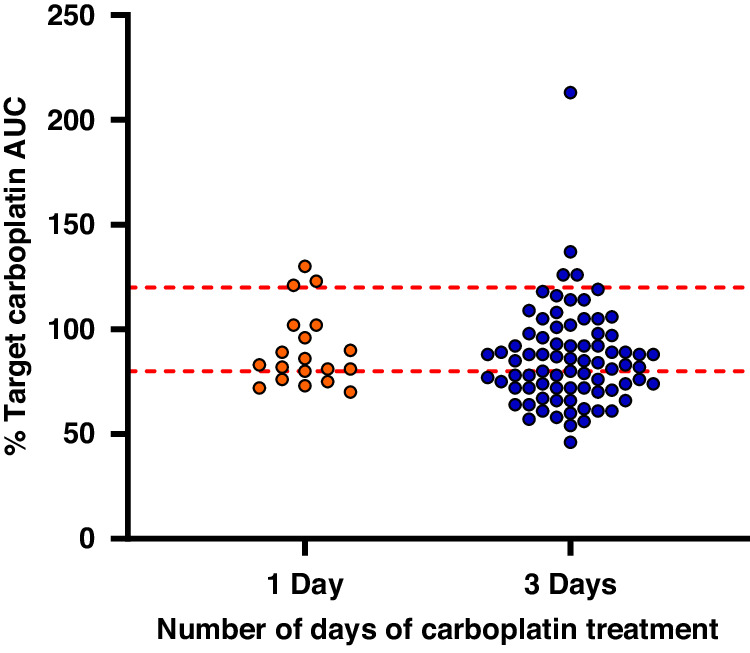
Predicted carboplatin drug exposures with protocol-based dosing regimens in the absence of TDM adaptive dosing. The single day treatment data show actual AUC values achieved across 19 cycles of treatment in 13 patients. For patients dosed over 3 days, the cumulative AUC values are predicted based on day 1 drug clearance and AUC from a total of 74 cycles of treatment in 29 patients, assuming no dose modification on day 3 of treatment. Red dashed lines show a 20% deviation from the defined target AUC.

The number of cycles of treatment on which TDM was utilised varied (range 1–6, median 3), with 25 patients being studied on at least 2 cycles. Starting doses across the 74 cycles of treatment studied varied from 4.1 to 6.9 mg/kg on day 1 of treatment. The UK protocol for treatment of children with retinoblastoma under 3 months recommends a starting carboplatin dose of 4.4 mg/kg to target an AUC of 5.2 mg/ml.min, with a recommendation to increase this to 6.6 mg/kg and 7.8 mg/ml.min, respectively, in the case of inadequate response. Children > 3 months received an initial dose of 100 mg/m^2^ on day 1 of treatment. Minor variations to these dosing recommendations are predominantly due to rounding of doses and changes in body weight between ordering and administering chemotherapy. Dose adjustments were made on day 3 to ensure that the target cumulative AUC was achieved. Based on the carboplatin clearance and AUC observed on day 1 of treatment, it was possible to accurately predict cumulative carboplatin exposures that would have been achieved over 3 days of treatment with protocol-based dosing regimens in the absence of TDM adaptive dosing. The predicted carboplatin exposure as a percentage of the target exposure varied markedly in this patient population, ranging from 46–213% as shown in Fig. 1. Without the adoption of a TDM approach to treatment, carboplatin exposures would have been ≥20% below the target cumulative AUC on 34/78 cycles of treatment (44%) and ≥20% above the target cumulative AUC on 4/78 cycles of treatment (5%).

**Fig. 2:**
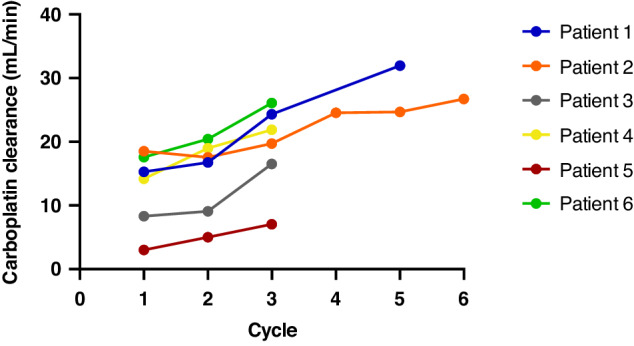
Carboplatin clearance values determined across multiple cycles of treatment in six patients aged between 3 weeks and 5 months of age.

For 13 patients who received carboplatin as a single day of treatment, drug exposures were determined across a total of 19 cycles of treatment. The actual AUC values observed on all cycles are shown in Fig. 1, with drug exposures achieved commonly falling outside the desired therapeutic window. The percentage of target AUC observed on cycle 1 of treatment ranged from 72 to 130%, with carboplatin exposures ≥20% below the target cumulative AUC on 6/19 cycles of treatment (32%) and ≥20% above the target cumulative AUC on 3/19 cycles of treatment (16%). Interestingly, the variation in drug exposure was comparable for subsequent cycles of treatment, even in patients where the carboplatin dose was modified based on the results from cycle 1 of treatment. This highlights the marked differences in carboplatin clearance that are frequently observed between cycles of treatment with these very young children. Figure 2 shows a clear trend towards an increase in carboplatin clearance across consecutive cycles of treatment in six patients aged between 3 weeks and 5 months of age, where data were available from between 3 and 6 treatment cycles.

**Fig. 3:**
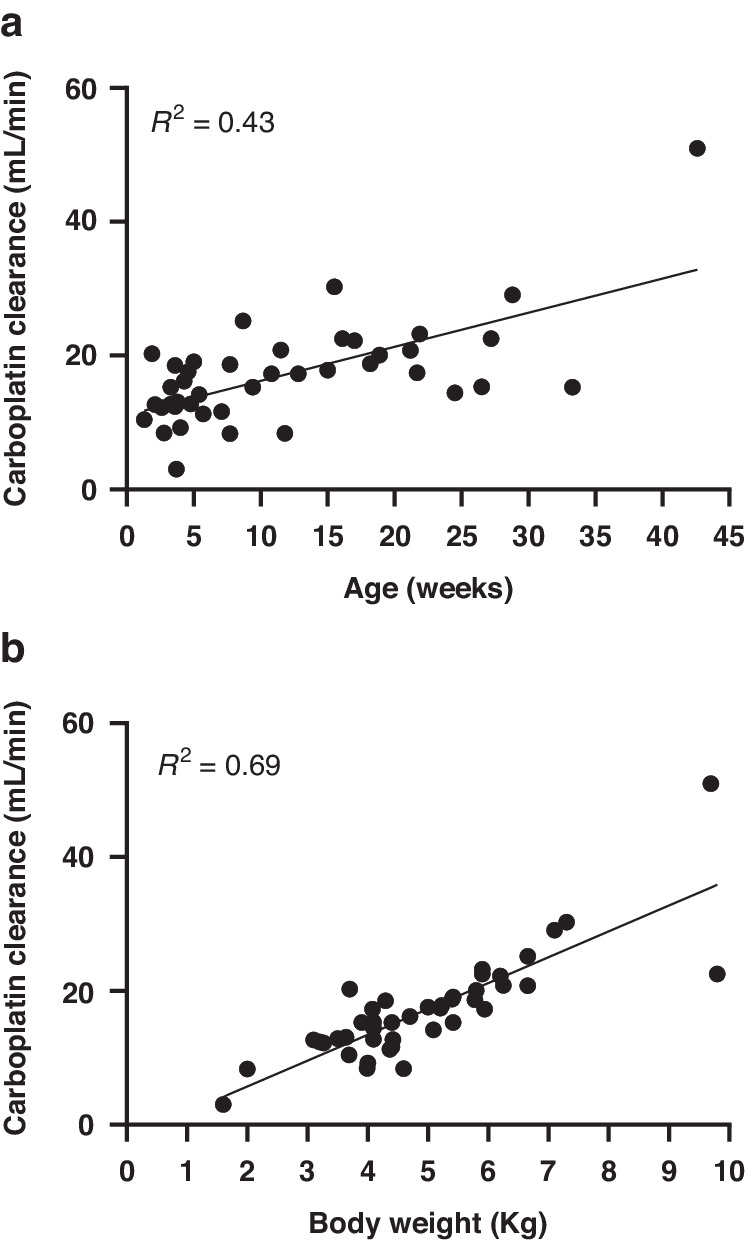
Carboplatin clearance determined on the first cycle of treatment for 43 children in the first year of life. Figure (**a**) shows the correlation with patient age and figure (**b**) shows the correlation with patient weight.

Figure 3a shows a moderate correlation between patient age and carboplatin clearance determined on the first cycle of treatment for 43 children aged less than one year of age. While Fig. 3b shows a stronger correlation between patient body weight and carboplatin clearance on the first cycle of treatment, this does not take into account the frequently marked changes in carboplatin clearance observed between treatment cycles shown in Fig. 2. This highlights the challenges in proposing an appropriate carboplatin dosing regimen that will result in the attainment of accurate drug exposures across multiple cycles of treatment in neonates and infants. While body weight-based dosing represents the most widely accepted approach for carboplatin treatment in this patient population, it fails to account for the large increases in drug clearance commonly observed across consecutive cycles of treatment, which therefore necessitate marked increases in drug dose.

### Efficacy

In total, 97% of patients showed a positive response at their first examination under anaesthetic (EUA) after either one or two cycles of chemotherapy, reflecting different standard examination intervals between centres. Of the 29 patients who had real time TDM, 25 patients received additional treatment for intra-ocular control or in the case of relapse / progression (defined as the presence of new or persistent areas of disease following the completion of systemic chemotherapy). 20 patients received local treatment with thermotherapy or cryotherapy and 5 required intra-arterial chemotherapy. Only one patient required a secondary enucleation. No patients required external beam radiotherapy. The time from the end of treatment of systemic chemotherapy to the present ranged from 18 months to 11 years and all patients are currently stable with ongoing ophthalmology and oncology surveillance of varying time frames.

### Toxicity

#### Ototoxicity

In total, 28 out of 29 patients (97%) had no change in hearing immediately after chemotherapy or during follow up. 1 patient (3%) had Boston Grade 1 bilateral sensorineural hearing loss at the end of treatment following a normal baseline assessment [25]. There was no progression of this over 3 years of repeated assessments (see case summary below for more details). The patient did not require any hearing aids. Two patients (7%) had pre-existing bilateral sensorineural hearing loss prior to chemotherapy. Both had confirmed 13q deletion syndrome.

#### Nephrotoxicity

The median baseline creatinine was 20 µmol/L (range 11–45 µmol/L). All results were within the expected physiological parameters based on the age of the patient. The median end of treatment creatinine was also 20 µmol/L (range 13–30 µmol/L) as shown in Table 2. 7 out of 29 (24%) patients had a GFR performed at the end of treatment – all results were within normal limits for the age of the patient. Two patients had absolute values < 70 ml/min/1.73 m^2^ which were repeated 12 months later and showed normal maturation of function.

**Table 2:** Summary of observed toxicities.

Toxicity	
Renal toxicity Baseline Creatinine (µmol/L)	
Median	20
Range	11–45
End of Treatment Creatinine	
Median	20
Range	13–30
Haematological toxicity Red Blood Cell transfusions	
% patients	45
Median	1
Range	1–3
Platelet transfusions	
% patients	7
Median	2.5
Range	2–3
Febrile episodes Febrile neutropenia	
% patients	21
Median	1
Range	0–1
Febrile non-neutropenia	
% patients	24
Median	1
Range	0–1

#### Haematological toxicity

A total of 13 out of 29 patients (45%) required a packed red cell transfusion due to a haemoglobin (Hb) of less than 70 g/L. The median number of transfusions was 1 (range 1–3). Only 2 out of 29 patients (7%) required platelet transfusions (median 2.5 transfusions, range 2–3). A total of 21% of patients (6 out of 29) had at least one episode of febrile neutropenia (defined as fever more than 38 °C with an absolute neutrophil count of ≤1.0 × 10^9^L) and 24% of patients (7 out of 29) also had at least one episode of non-neutropenic fever (defined as fever more than 38 °C with an absolute neutrophil count of >1.0 × 10^9^L). There were no patients with occult septic episodes and no patients were admitted to paediatric intensive care. No cycles of chemotherapy were delayed due to prolonged myelosuppression in the patients who underwent TDM guided dosing. There was no statistical difference between AUC targeted (5.2 v 7.8 mg/mL.min) and incidence of febrile neutropenia (P value = 0.88). There was no correlation between AUC and need for transfusion as the transfusions were in patients with a targeted AUC of 5.2 mg/mL.min.

#### Notable cases

We have chosen four patients to highlight some of the interplay between drug exposures achieved using TDM, toxicity observed and efficacy of treatment. All four patients were born full term and were treated with a combination of carboplatin, etoposide and vincristine.

### Case 1

The patient was diagnosed with unilateral retinoblastoma at less than 4 weeks of age. They had a normal baseline hearing test. There was no family history of hearing loss and there were no parental concerns regarding hearing. The end of treatment hearing assessment showed Boston Grade 1 ototoxicity which has remained stable over time. The child is developing well and appropriately responding to sounds. If non-TDM guided dosing had been used, the cumulative carboplatin dose for the first three cycles would have been 900 mg/m^2^. Using TDM-guided dosing, the cumulative carboplatin dose required to achieve the target cumulative AUC was reduced to 755 mg/m^2^. He had also received aminoglycosides (amikacin) during his chemotherapy treatment for an episode of bacteraemia. Either the carboplatin or amikacin could have been responsible for the ototoxicity. If the carboplatin was responsible, given the dose reduction using TDM, it is possible that the patient may have had worse ototoxicity if non-TDM guided dosing was used.

### Case 2

This patient was 3 weeks and 4 days old at the start of treatment for bilateral retinoblastoma and achieved a good response to chemotherapy following a marked dose increase based on a TDM dosing approach. There was no need for additional tumour directed therapy (other than laser and cryotherapy). The cumulative carboplatin dose received was 2850 mg/m^2^, whereas a cumulative dose of 1800mg/m^2^ would have been received with non-TDM guided dosing. The patient required 3 packed red cell transfusions and 2 platelet transfusions during treatment, all given prior to the third cycle of chemotherapy. The mean dose for the first two cycles when transfusions were required was 336 mg/m^2^ versus 545 mg/m^2^ for the final four cycles when transfusions were not required. This case highlights that a marked increase in carboplatin dose was required based on TDM without which the patient may not have achieved such a good response. This may have led to a requirement for further therapy. This increased dose did not lead to increased toxicity.

### Case 3

This patient commenced chemotherapy at 10 days of age for a unilateral (genetic) retinoblastoma. They required all 6 cycles of chemotherapy to bring their tumour under control, despite receiving a markedly increased cumulative dose of carboplatin based on a TDM dosing approach. The cumulative carboplatin dose received was 2500 mg/m^2^, as compared to 1800 mg/m^2^ if a standard body surface area-based dosing approach had been implemented. The patient required 3 packed red cell transfusions in the first 3 cycles of chemotherapy. The mean dose for the first three cycles when transfusions were required was 320 mg/m^2^, versus 515 mg/m^2^ for the final three cycles when transfusions were not required. Given the large difference in carboplatin exposure if non-TDM dosing had been used, and the fact that all 6 cycles of chemotherapy were required for tumour control, it is possible that second line systemic or IA chemotherapy may have been required without TDM dosing.

### Case 4

This patient commenced chemotherapy at 4 weeks of age for a unilateral retinoblastoma. Initial carboplatin treatment utilised TDM dosing with a mean dose of 280 mg/m^2^ for the first 2 cycles. The patient required one packed red cell transfusion after the first cycle and also had one episode of uncomplicated febrile neutropenia. There was an excellent response to chemotherapy after two cycles with almost complete regression of the tumour. For the third cycle of chemotherapy, the patient reverted to standard per protocol dosing (cumulative dose of 13.2 mg/kg which for this patient was equivalent to 225 mg/m^2^). This was equivalent to a 20% reduction based on the dose delivered during previous cycles of chemotherapy. The patient had a dramatic recurrence of tumour following this cycle. There was an attempt to salvage the eye with a further cycle of TDM-guided chemotherapy and one course of secondary intra-arterial chemotherapy but tumour control was not achieved and the patient proceeded to have a secondary enucleation. It is unclear whether this recurrence would have occurred if the patient had continued with TDM guided dosing given the higher dosing recommended for this patient.

## Discussion

Retinoblastoma is the most common intra-ocular malignancy in childhood and has an excellent survival rate of greater than 95% at 5 years in developed countries such as the UK [26]. However, carboplatin is associated with potentially severe side effects including ototoxicity and nephrotoxicity whilst etoposide is associated with a risk of secondary malignancies [1, 6–9, 14, 27]. Retinoblastoma can present in very young infants which poses known challenges for the delivery of systemic chemotherapy, with a variety of methods adopted [18]. This report outlines the UK experience of utilising carboplatin real-time TDM in neonates and infants with retinoblastoma, with adaptive dosing to allow uniformity in dosing based on AUC in response to developmental changes in physiology in the first few months of life.

We have shown in previous studies that this approach to treatment is feasible and reproducible. It is now standard practice in the UK for infants under the age of 3 months to receive a fractionated dose of carboplatin over 3 days, with adaptive dosing carried out on the third day to ensure that the target cumulative drug exposure is achieved [22, 28]. This approach avoids the attainment of drug exposures outside of the therapeutic window, which may be associated with increased drug toxicity or reduced efficacy.

In the current study we provide real world TDM data generated from a total of 29 patients and 74 cycles of carboplatin treatment. These data show that without the adoption of a TDM approach to treatment, the majority of infants achieve drug exposures below the target cumulative AUC when dosed based on either body weight or body surface area based calculations. Approximately one-third of these patients are likely to achieve exposures ≥20% below the cumulative target AUC. This could increase the risk of inadequate treatment for this patient population, potentially leading to an increased need for additional subsequent treatment and/or treatment failure. Considering 90% of our patient cohort had genetic disease, highlighting the heritable nature of this condition and the additional risks this poses including second malignant neoplasms, adequately dosing these patients is of the utmost importance.

Our finding of consistent under-dosing based on current protocol dosing recommendations is consistent with recently published data suggesting carboplatin doses of 6 mg/kg and 9 mg/kg to target AUC values of 5.2 mg/mL.min and 7.8 mg/mL.min, respectively, in neonates and infants <10 kg. These updated dosing recommendations will now be adopted in the UK.

For an additional 13 patients, carboplatin clearance was calculated retrospectively following a single dose of carboplatin with the aim to guide future treatment cycles. However, due to the physiological changes between cycles, resulting in marked changes in carboplatin clearance, this approach cannot be recommended and should only be considered in circumstances where real-time monitoring is not feasible. A key finding of the current study is the change in carboplatin clearance observed with sequential cycles of treatment during the first weeks and months of life. These marked and sometimes rapid changes in drug clearance can necessitate the need for relatively large increases in carboplatin dose between treatment cycles, which are far more dramatic than the dose increases that would be made based on relatively small changes in body weight or body surface area. These changes in drug clearance between cycles of treatment are likely to be related to marked changes in kidney maturation observed early in life and cannot be predicted or accounted for thus highlighting the importance of utilising a TDM approach to dosing.

In terms of clinical response, 97% of patients studied showed a positive response at their first examination under anaesthetic (EUA) after either one or two cycles of chemotherapy. A total of 17% of patients in our cohort required secondary intra-arterial chemotherapy and only one patient had a secondary enucleation. No patients received external beam radiotherapy. This compares favourably with other historical cohorts treated with systemic chemotherapy where 37–47% of patients required enucleation and 0–27% received external beam radiotherapy [11, 14, 29]. Some of these differences may be explained by the stage of the tumour at diagnosis and newer techniques including intravitreal chemotherapy, which were not available in some historical cohorts. However, we believe that the higher doses of carboplatin delivered to many patients based on a TDM dosing approach, is also likely to have contributed to the excellent clinical outcomes observed in these patients.

The major concern with increased doses of carboplatin in young infants is the potential for an elevated incidence of toxicity which has the potential to have lifelong consequences. This is particularly pertinent when we consider these children are already at risk of visual impairment from their underlying disease. There are specific concerns about ototoxicity, nephrotoxicity and increased myelosuppression. The use of platinum agents such as carboplatin has previously been shown to be associated with an increased risk of ototoxicity. The incidence of ototoxicity in children with retinoblastoma ranges from 0–20% with the largest published series showing a rate of hearing impairment of 4.5% [6–10]. Only one (3%) patient in our cohort had hearing impairment following their chemotherapy, suggesting that increased carboplatin dosing based on a TDM approach does not lead to an increased risk of ototoxicity in this patient group. A recently published systematic review and meta-analysis on ototoxicity in patients treated with platinum based chemotherapy found the incidence of carboplatin induced ototoxicity to be 7.4% in children <5 years of age at diagnosis. Furthermore, and of particular relevance to our results, they showed no relationship between overall dose of platinum agent and likelihood of hearing loss (meta-regression model) [30].

The patients who underwent real time TDM on this study had an encouraging toxicity profile overall, with no paediatric intensive care admissions and no deaths due to sepsis or toxicity. This fits with previously published data suggesting that the combination of carboplatin, etoposide and vincristine is generally well tolerated in this clinical setting. Carboplatin is known to be associated with nephrotoxicity although this is less severe than reported with some other chemotherapeutic agents, particularly cisplatin. In our cohort, we saw no reduction in GFR in those patients where this was formally measured, and the mean creatinine before and after treatment was identical for the whole cohort. There is conflicting evidence about the impact of carboplatin on GFR following treatment. One UK based cohort identified a mean fall in GFR of 22 ml/min/1.73 m^2^ in patients receiving carboplatin, although the doses used in this cohort were significantly higher than in the current study (median dose 2590 mg/m^2^; range 1364–7133 mg/m^2^). All patients in this cohort still had normal renal function based on GFR [4]. A nationwide German surveillance study did not identify any change in GFR after treatment with platinum agents despite most patients receiving other nephrotoxic drugs (such as ifosfamide), abdominal radiotherapy or both [31]. One patient in our cohort had a GFR of <70 ml/min/1.73 m^2^. It is important to remember the rapidly changing physiology in young children with a relatively rapid increase in GFR within the first two years of life. A GFR of <70 ml/min/1.73 m^2^ is within the acceptable normal range for a child at that age and increased appropriately when repeated serially [32].

A total of 45% of patients in our cohort received at least one packed red cell transfusion whilst only 7% required a platelet transfusion. This compares to data published from Germany in children with retinoblastoma receiving chemotherapy (median age at first cycle of chemotherapy 6 months), which showed that 21% of patients required a platelet transfusion and only 9% of patients required packed red cell transfusion, and a paper by Munier et al (median age at first cycle of chemotherapy 19.2 months) in which 13% of patients required packed red cell transfusion and 22% required a platelet transfusion [5, 14]. While some of these differences may be due to the increased carboplatin dosing associated with TDM, given that the need for platelet transfusions was lower than both cohorts, we believe that some of the differences may also be explained by repeated blood sampling in smaller infants with a lower circulating blood volume. This would be partly supported by the fact that the two patients who received 3 packed red cell transfusions were both neonates at the onset of chemotherapy. It is further supported by the fact that the patients who required transfusions received them in the first three cycles of chemotherapy when they were youngest and not in subsequent cycles when they may have received higher doses of chemotherapy. This is further corroborated by the significantly younger age of our cohort than the two discussed.

## Limitations

The data presented here is from a retrospective review of patients in the UK who underwent TDM to support their treatment for retinoblastoma. The retrospective nature of these data means that not all data were available for analysis and also risks some element of bias. Similarly, as there were no mandated investigations included in this retrospective analysis, only 7 patients had formal end of treatment GFR monitoring and not all patients had routine audiology surveillance. The rarity of retinoblastoma means that we have accrued the data presented in the current study from 29 patients studied over a period of more than fifteen years. Some treatments for retinoblastoma (intra-arterial and intra-vitreal chemotherapy) and supportive treatments will have changed over this time (including blood transfusion thresholds) which may impact on the applicability of the results to a certain degree.

## Conclusions

The current study highlights the benefit of delivering carboplatin to infants with retinoblastoma using a real-time TDM approach. This avoids the likelihood that a significant number of patients achieve drug exposures ≥20% above or below the target cumulative AUC. The data generated indicate that utilising a TDM approach to treatment results in the majority of patients receiving higher doses of carboplatin than if they had been dosed by body weight or surface area based methods. Despite this increased dose, the toxicity observed is in keeping with other published cohorts and most notably there is no increase in ototoxicty, which is arguably the most devastating late effect in a population of children at risk of visual deficit. This is a significant finding bearing in mind the very young age of the patient population studied, with the vast majority of patients treated in the first weeks of life, a patient population at high risk of experiencing ototoxicity following carboplatin treatment. We believe that these findings provide strong evidence that TDM-based dosing should be implemented as standard of care for all infants less than 6 months of age receiving systemic carboplatin treatment for retinoblastoma. Where a TDM approach to treatment is not possible, updated guidelines to be published this year will recommend carboplatin doses of 6 mg/kg and 9 mg/kg for targeting AUC values of 5.2 mg/mL.min and 7.8 mg/mL.min, respectively, in neonates and infants <10 kg [33].

## Data Availability

The data that support the findings of this study are available on request from the corresponding author.
